# The Evolving Landscape of microRNAs in Cholangiocarcinoma and Pancreatic Cancer

**DOI:** 10.3390/diagnostics15182285

**Published:** 2025-09-09

**Authors:** Andrada Ozana Schneider, Sabrina Birsan, Paula Anderco, Cristian Ichim, Samuel Bogdan Todor, Horatiu Dura, Radu Fleacă, Adrian Boicean

**Affiliations:** Faculty of Medicine, Lucian Blaga University of Sibiu, 550024 Sibiu, Romania; andradaozana.schneider@ulbsibiu.ro (A.O.S.); cristian.ichim@ulbsibiu.ro (C.I.); samuelbogdant@gmail.com (S.B.T.); horatiu.dura@ulbsibiu.ro (H.D.); radu.fleaca@ulbsibiu.ro (R.F.); adrian.boicean@ulbsibiu.ro (A.B.)

**Keywords:** microRNA, cholangiocarcinoma, pancreatic ductal adenocarcinoma, chemoresistance, biomarkers, targeted therapy

## Abstract

Cholangiocarcinoma (CCA) and pancreatic ductal adenocarcinoma (PDAC) are aggressive malignancies with limited therapeutic options and poor prognoses. In recent years, microRNAs (miRNAs) have gained attention as key molecular regulators involved in tumor progression, chemoresistance, and metastasis. This review explores the diagnostic, prognostic, and therapeutic potential of miRNAs in CCA and PDAC, emphasizing their shared and distinct molecular pathways and their utility in the context of precision oncology. Several dysregulated miRNAs, most notably miR-21 and miR-155, are overexpressed in both cancers and contribute to activation of oncogenic pathways such as PI3K/AKT signaling, epithelial–mesenchymal transition, and inflammatory cascades. miR-21, in particular, is associated with resistance to gemcitabine and cisplatin. In contrast, tumor-suppressive miRNAs such as miR-34a and miR-145 are often downregulated, and their restoration using synthetic mimics has demonstrated promising antitumor effects in preclinical studies. Moreover, circulating miRNAs show potential as non-invasive biomarkers for early detection and disease monitoring. Advanced delivery platforms, including nanoparticles and exosome-based systems, are being developed to improve the stability and tumor specificity of miRNA-based therapeutics. miRNAs represent a promising class of molecules in the diagnosis, stratification, and treatment of CCA and PDAC. Their dual role as biomarkers and therapeutic agents positions them at the intersection of molecular pathology and personalized medicine. Further multicenter clinical trials and mechanistic studies are needed to validate their clinical applicability and to refine delivery strategies for targeted miRNA modulation.

## 1. Introduction

Bile duct cancers (BDC) constitute a rare and heterogeneous group of malignancies arising from the epithelial lining of the biliary tree. Anatomically, they are classified into intrahepatic bile duct cancer, which originates within the hepatic parenchyma, and extrahepatic bile duct cancer, which develops beyond the liver. The extrahepatic category is further subdivided into perihilar and distal subtypes, according to the tumor’s anatomical relationship to the hepatic hilum [1,2]. Histologically, intrahepatic cholangiocarcinoma (CCA) can be subdivided into two major subtypes. The small-duct type intrahepatic CCA typically arises from peripheral bile ductules or the canals of Hering and is frequently associated with chronic liver disease or hepatitis-related injury. This subtype is characterized by the presence of smaller glandular structures and shows a higher frequency of genetic alterations, particularly IDH1/2 mutations and FGFR2 fusions. In contrast, the large-duct type intrahepatic CCA originates from the larger intrahepatic bile ducts, exhibits molecular and morphological similarities to perihilar CCA, and is commonly linked to chronic cholangitis, primary sclerosing cholangitis, or hepatolithiasis. This variant often demonstrates mucin production and is more frequently associated with KRAS and TP53 mutations [1,2,3]. Each subtype of BDC exhibits distinct molecular profiles, etiological factors, and epidemiological trends [1,2]. Histologically, both intrahepatic and extrahepatic CCA are most commonly adenocarcinomas, accounting for the vast majority of cases. Rare variants, such as adenosquamous, mucinous, or clear-cell carcinomas, have been described but remain exceedingly uncommon and lack specific therapeutic implications [4]. Mirroring the clinical course of pancreatic ductal adenocarcinoma, BDC is characterized by a dismal prognosis. In advanced stages, the median overall survival generally ranges between 6 and 12 months, largely attributable to delayed diagnosis, inherent tumor aggressiveness, limited treatment modalities, and the frequent emergence of chemoresistance [1,2,3].

Current international guidelines endorse surgical resection followed by adjuvant chemotherapy as the sole potentially curative approach for BDC [5,6,7]. Nonetheless, curative resection is achievable in only approximately 30% of patients, primarily due to advanced disease at the time of diagnosis. Even among those undergoing resection, long-term survival remains suboptimal, with merely 20–40% of patients deriving substantial clinical benefit [7,8,9]. For the majority of individuals presenting with unresectable or metastatic disease, first-line systemic therapy typically involves a combination of gemcitabine and cisplatin [10,11,12,13]. While this regimen represents the current therapeutic standard, it offers only modest efficacy, marked by limited tumor response rates and frequent disease progression, ultimately resulting in poor clinical outcomes [14,15].

In recent years, considerable attention has been directed toward the development of novel therapeutic strategies and the identification of robust biomarkers for diagnosis, prognosis, and therapeutic response prediction in both BDC and pancreatic cancer [16,17,18]. The emergence of high-throughput genomic and transcriptomic technologies has greatly advanced the discovery of molecular targets, thereby paving the way for precision oncology and targeted interventions in these malignancies [19,20]. Among the most compelling molecular regulators identified are microRNAs (miRNAs), short, non-coding RNA molecules that exert post-transcriptional control over gene expression. These miRNAs are critically involved in a spectrum of cellular processes, including proliferation, apoptosis, differentiation, invasion, and metastasis.

In CCA, miRNAs can act either as oncogenes (oncomiRs) or tumor suppressors, thereby contributing to tumor initiation, progression, therapeutic resistance, and metastatic dissemination [20,21,22,23]. Dysregulated miRNA expression in CCA has been implicated in the modulation of multiple oncogenic signaling cascades, positioning miRNAs as promising candidates for both non-invasive biomarkers and therapeutic targets. Comparable aberrations have been documented in pancreatic ductal adenocarcinoma (PDAC), where miRNAs similarly influence disease trajectory and response to treatment [23,24,25].

Notably, CCA and PDAC exhibit overlapping miRNA-regulated molecular pathways, such as KRAS, PI3K/AKT, TGF-β, and Notch signaling, highlighting shared oncogenic mechanisms and potential convergent therapeutic vulnerabilities. Nevertheless, significant differences persist in the expression profiles and functional consequences of individual miRNAs between these two malignancies, reflecting their distinct cellular origins and tumor microenvironments [24,25,26,27]. This narrative review aims to delineate both the commonalities and divergences in miRNA-driven molecular regulation in BDC and pancreatic cancer, with a particular focus on identifying actionable biomarkers and informing treatment stratification in these biologically aggressive tumors.

## 2. Materials and Methods

A comprehensive literature search was conducted in the PubMed and Scopus databases to identify studies addressing the role of microRNAs (miRNAs) in cholangiocarcinoma (CCA) and pancreatic ductal adenocarcinoma (PDAC). The search covered all publications from database inception until 31 December 2024, using combinations of the following keywords with Boolean operators: microRNA, miRNA, cholangiocarcinoma, bile duct cancer, bile duct carcinoma, pancreatic cancer, and pancreatic ductal adenocarcinoma. Eligible studies included both original research articles, encompassing preclinical, translational, and clinical investigations, and review papers that examined miRNA expression patterns, molecular signaling pathways, or clinical implications in CCA or PDAC. Studies focusing on other biliary tract malignancies or pancreatic tumor types were excluded, unless they provided separate analyses or extractable data relevant to CCA or PDAC.

Screening was performed in two stages: titles and abstracts were first evaluated to exclude clearly irrelevant records, followed by full-text review of potentially eligible articles. Additionally, the reference lists of key publications and reviews were manually screened to identify further studies not captured by the primary database search.

From the eligible studies, data were narratively extracted with emphasis on the identity and expression profiles of miRNAs (whether upregulated, downregulated, or context-dependent), their main molecular targets and associated signaling pathways, the biological source analyzed (such as tissue, serum, plasma, bile, or urine) and the detection methods applied (qRT-PCR, microarray or next-generation sequencing). Particular attention was given to the clinical significance of reported findings, including diagnostic, prognostic, and therapeutic implications, as well as outcomes observed in preclinical and clinical settings. Two reviewers independently conducted the screening and full-text evaluation. Any discrepancies were resolved through discussion, and all studies meeting the inclusion criteria were integrated into the narrative synthesis.

## 3. Molecular Changes in Cholangiocarcinoma

miRNAs have emerged as pivotal molecular regulators in the diagnosis, prognosis, and treatment of CCA, owing to their post-transcriptional control of gene expression and involvement in key oncogenic signaling pathways [21,22,23,24,25,26,27,28,29,30]. Ongoing therapeutic efforts are increasingly focused on harnessing the dual functionality of miRNAs, which may act either as tumor suppressors or as oncomiRs, depending on the cellular context. Notably, intrahepatic and extrahepatic CCA exhibit distinct molecular and miRNA profiles. iCCA is frequently characterized by IDH1/2 mutations, FGFR2 fusions, and upregulation of miR-21 and miR-31, whereas eCCA more often shows KRAS and TP53 mutations, SMAD4 loss, and dysregulation of the miR-200 family and miR-192, which drive epithelial–mesenchymal transition and metastasis [4].

One promising therapeutic avenue involves the use of miRNA mimics, synthetic RNA constructs engineered to restore the expression and function of downregulated tumor-suppressive miRNAs. A prototypical example is miR-34a, a well-characterized tumor suppressor, whose synthetic analog miR-34a mimic (MRX34) has entered clinical trials for several malignancies, including CCA [30,31,32,33,34,35]. These mimics aim to reinitiate tumor-suppressive programs, thereby promoting apoptosis and inhibiting neoplastic progression.

Conversely, several miRNAs are overexpressed in CCA and function as oncogenes. Among the most extensively studied is miR-21, which is consistently upregulated in both tumor tissues and circulating fluids of patients with CCA [31,32,33,34]. miR-21 facilitates tumor growth, survival, and invasion by targeting a range of tumor suppressor genes, including PTEN, PDCD4, and PTPN14 [35,36,37]. It also promotes epithelial–mesenchymal transition (EMT), a critical step in metastasis, partly through suppression of 15-hydroxyprostaglandin dehydrogenase [30,33,34,35,36,37,38,39,40,41]. Elevated miR-21 expression has been strongly correlated with adverse clinical outcomes, including reduced overall survival, thus positioning it as a compelling biomarker for both diagnostic and prognostic purposes [42,43,44,45,46,47,48,49,50,51,52,53,54,55,56,57,58,59,60,61,62,63,64,65]. Among the most promising non-invasive biomarkers for CCA are circulating miRNAs, such as miR-21 and miR-192, detectable in serum, plasma, and bile. These circulating miRNAs not only reflect disease status but are also implicated in tumor progression and resistance to therapy [66,67,68,69,70,71,72,73,74,75,76,77,78,79]. Their diagnostic utility is reinforced by their stability, accessibility, and functional relevance.

From a therapeutic standpoint, two major strategies are currently under investigation: inhibition of oncogenic miRNAs and restoration of tumor-suppressive miRNAs. Anti-miR-21 therapies aim to neutralize the tumor-promoting activity of miR-21, thereby impeding proliferation, metastasis, and chemoresistance. In contrast, miR-34a-based therapies, such as MRX34, seek to re-establish tumor-suppressive signaling pathways by reinstating the expression of silenced miRNAs, ultimately inducing apoptosis and suppressing tumor growth [21,79,80,81,82,83,84,85]. A comprehensive overview of miRNAs implicated in CCA progression and their potential therapeutic applications is summarized in Table 1, underscoring their relevance as emerging tools in the personalized management of CCA.

From a therapeutic perspective, anti-miR-21 inhibitors have demonstrated significant potential in preclinical models by reducing tumor growth and enhancing the chemosensitivity of CCA cells to standard agents such as gemcitabine and cisplatin [46,47]. Beyond miR-21, other oncogenic miRNAs also contribute to the aggressive phenotype of CCA. Notably, miR-221/222 promote cell cycle progression and inhibit apoptosis by targeting critical regulatory genes, including p27 and PTEN, while miR-155 has been strongly associated with inflammation-induced carcinogenesis, reinforcing the well-established link between chronic inflammation and bile duct tumorigenesis [1,3,4,5,48,49,50].

Conversely, a range of tumor-suppressive miRNAs are frequently downregulated in CCA and exert protective functions against tumor initiation and progression. miR-34a, in addition to its therapeutic relevance, suppresses cellular proliferation and promotes apoptosis through inhibition of oncogenic pathways involving Notch and Bcl-2. The miR-200 family plays a pivotal role in suppressing EMT, thereby limiting metastatic dissemination, whereas miR-145 counteracts tumor growth by targeting key oncogenes such as MDM2 and c-Myc. Reduced expression of these miRNAs in patient samples has been consistently correlated with advanced disease stage, increased metastatic potential, and poorer clinical outcomes [51,52,53,54,55,56,57].

Therapeutically, antagomiRs, chemically engineered oligonucleotides designed to silence oncogenic miRNAs, are under active development. Among these, anti-miR-21 therapy represents a leading candidate, targeting the persistently upregulated miR-21, which plays a central role in driving proliferation, metastasis, and chemoresistance in CCA [58,59,60,61,62,63,64]. Inhibition of miR-21 has been shown to restore the expression of tumor suppressors and to sensitize tumor cells to cytotoxic agents, providing a compelling rationale for its use in combination with existing chemotherapeutic regimens [28,48].

To enhance the clinical utility of both miRNA mimics and inhibitors, advanced delivery systems are being investigated to address key challenges related to molecular stability, targeted delivery, and off-target effects. Novel platforms, such as nanoparticles, liposomes, and exosome-based carriers, have been engineered to shield therapeutic miRNAs from enzymatic degradation in circulation and to facilitate selective accumulation in tumor tissues. Among these, exosome-mediated delivery is particularly promising, owing to its inherent biocompatibility, low immunogenicity, and natural tropism for malignant cells, making it an ideal candidate for precision-targeted therapies in CCA [50,65,66,67,68,69,70,71,72,73,74,75,76,77].

Future directions in miRNA-based therapy for CCA are progressing rapidly, with several ongoing clinical trials evaluating the safety, tolerability, and efficacy of these innovative strategies [74,75,76,77,78,79,80,81,82,83]. The integration of miRNA-targeted approaches with conventional chemotherapy, particularly gemcitabine and cisplatin, holds substantial promise for enhancing treatment responses and overcoming chemoresistance, which remains a major therapeutic hurdle in advanced-stage CCA [83,84,85].

Among the most extensively characterized oncogenic microRNAs, miR-21 has been closely linked to chemoresistance in CCA. Notably, miR-21 mediates resistance to Hsp90 inhibitors by directly targeting DNAJB5, a key co-chaperone involved in protein folding and the cellular stress response. Preclinical studies have demonstrated that inhibition of miR-21 in CCA cell lines restores sensitivity to Hsp90 blockade, thereby representing a promising strategy to circumvent this resistance mechanism [33,37,38]. Although miRNA-based therapies are still largely confined to preclinical and early-phase clinical evaluation, the therapeutic landscape for advanced CCA has evolved considerably in recent years, with several molecularly targeted agents gaining regulatory approval. One such agent is pemigatinib, a selective fibroblast growth factor receptor 2 (FGFR2) inhibitor, approved for patients with FGFR2 gene fusions or rearrangements, a genomic alteration observed in a subset of intrahepatic cholangiocarcinoma cases. In pivotal clinical trials, pemigatinib demonstrated an overall response rate of 36%, with a subset of patients achieving durable responses exceeding six months [74,75,76,77,78,79].

Another notable targeted therapy is ivosidenib, approved for patients with isocitrate dehydrogenase 1 (IDH1)-mutated CCA. Ivosidenib acts by inhibiting the mutant IDH1 enzyme, thereby reducing intracellular levels of the oncometabolite 2-hydroxyglutarate (2-HG). This reduction in 2-HG facilitates malignant cell differentiation and slows tumor progression [77,78,79,80,81,82]. Together, these agents exemplify the progress toward precision oncology in CCA, offering biomarker-driven treatment options that enhance therapeutic efficacy and patient outcomes. The successful clinical implementation of FGFR2 and IDH1 inhibitors underscores the feasibility and therapeutic value of molecularly tailored interventions in the management of cholangiocarcinoma [64,65,66,67,68].

## 4. miRNAs in Pancreatic Cancer

miRNAs are increasingly recognized as central regulators in the pathogenesis of PC, particularly PDAC, the most prevalent and aggressive histological subtype. These small, non-coding RNAs orchestrate diverse oncogenic processes, including cellular proliferation, metastasis, chemoresistance, and immune evasion, thereby emerging as both diagnostic biomarkers and therapeutic targets [55,56,57,58,59,60,61,62,63,64,65,66,67,68]. Among these, miR-370 has garnered significant attention due to its context-dependent behavior. Depending on the tumor microenvironment and molecular targets, miR-370 may function either as a tumor suppressor or as an oncogene [1,61]. This dualistic nature exemplifies the functional plasticity of miRNAs and underscores the necessity for molecular stratification when considering miRNA-targeted therapies [67,68]. In PDAC subtypes where miR-370 is downregulated, its tumor-suppressive activity can be therapeutically restored using miR-370 mimics. These synthetic constructs have shown promise in reversing chemoresistance, particularly to gemcitabine, by promoting apoptosis and inhibiting key oncogenic pathways [74,75,76,77,78,79]. Conversely, in contexts where miR-370 is overexpressed and exerts oncogenic effects, anti-miR-370 agents may be employed to suppress its activity, thus limiting tumor progression.

Mechanistically, miR-370 exerts tumor-suppressive functions by inhibiting FOXM1 and Wnt/β-catenin signaling, two pathways frequently hyperactivated in PDAC and associated with poor clinical outcomes. Additionally, miR-370 impedes EMT via direct targeting of TGFBR2, thereby attenuating metastatic potential. Its ability to sensitize tumor cells to gemcitabine further enhances its therapeutic relevance [62,63,64,65,66,67,68,69,70,71,72,73,74]. However, in specific molecular contexts, miR-370 adopts an oncogenic role, promoting tumor progression by targeting tumor suppressors such as PTEN and TP53 and by modulating cytokine expression and inflammatory signaling within the tumor microenvironment [74,75,76,77,78,79].

Another miRNA of notable significance is miR-361, which similarly displays context-specific functions. In most PDAC tissues and cell lines, miR-361 is downregulated and acts as a tumor suppressor. Functional studies have shown that miR-361 overexpression reduces cell viability, induces apoptosis, and inhibits migration by targeting the MAPK/JNK signaling pathway [62,63,64,65]. In contrast, the miR-361-3p isoform has demonstrated oncogenic properties, particularly through the inhibition of DUSP2, which leads to ERK pathway activation and enhances EMT, invasion, and liver metastasis in vivo [61,62,63,64,65].

Therapeutically, miRNA-based strategies are advancing rapidly. One major approach involves miRNA mimics, synthetic analogs that restore the expression of tumor-suppressive miRNAs. For example, MRX34 has undergone clinical trials due to its capacity to suppress tumor proliferation. Conversely, antagomiRs are employed to silence overexpressed miRNAs such as miR-21, which has shown efficacy in reducing chemoresistance and enhancing the effectiveness of cytotoxic agents across multiple malignancies, including PDAC [63,64,65,66,67,68,69,70,71]. Despite their therapeutic promise, the clinical translation of miRNA-based treatments faces key challenges related to stability, delivery, and specificity. To overcome these obstacles, advanced delivery platforms, including nanoparticles, liposomes, and exosome-based systems, have been developed. These vehicles enhance miRNA stability in circulation, improve bioavailability, and enable targeted delivery to tumor tissues while minimizing off-target effects. Exosome-based carriers, in particular, offer unique advantages such as low immunogenicity, high biocompatibility, and natural tropism toward malignant cells, positioning them as frontrunners for precision medicine in pancreatic cancer [61,62,63,64,65].

Beyond their therapeutic implications, miRNAs also hold substantial value as non-invasive biomarkers. Circulating miRNAs, notably miR-21 and miR-192, detectable in blood or bile, have shown potential for early diagnosis, prognosis assessment, and treatment monitoring. Their expression profiles reflect underlying disease biology and may facilitate patient stratification and personalized treatment selection [66,67,68].

Looking forward, numerous clinical trials are underway to evaluate the safety and efficacy of miRNA-based interventions across various cancer types, including PDAC. The integration of miRNA therapeutics with conventional chemotherapies, such as gemcitabine, represents a promising strategy to enhance treatment response and to overcome the pervasive challenge of drug resistance in pancreatic cancer [64,65,66,67,68]. These miRNAs suppress tumorigenesis, and their downregulation contributes to cancer progression (Table 2).

Pancreatic cancer is often diagnosed late due to a lack of early symptoms. miRNAs in blood, urine, and pancreatic juice serve as non-invasive biomarkers for early detection (Table 3).

A growing number of studies have focused on circulating miRNA panels, particularly miR-21, miR-155, and miR-196a, detected in peripheral blood as non-invasive biomarkers for the early detection, prognostic stratification, and therapeutic monitoring of PDAC [62,63,64]. For example, serum-based miRNA profiling in newly diagnosed PDAC patients, both at baseline and during treatment, has demonstrated the ability to discriminate between malignant and healthy states, while also correlating with therapeutic response. These findings hold promise for the development of personalized, miRNA-guided treatment strategies [74,75,76].

Beyond blood-based assays, urinary miRNA signatures have also emerged as reliable diagnostic tools. Notably, several urinary miRNA-based assays have demonstrated superior sensitivity and specificity compared to the conventional CA19-9 biomarker, especially for detecting early-stage PDAC. This highlights the translational potential of miRNA-based diagnostics in routine clinical screening [65,66,67,76].

On the therapeutic front, miRNA-based treatments are currently undergoing preclinical and early clinical investigation. MRX34, a synthetic mimic of the tumor-suppressive miR-34a, entered clinical trials as the first miRNA-based cancer therapy. However, the trial was halted due to immune-related adverse events, underscoring the importance of optimizing delivery mechanisms and improving patient selection criteria [62,63,64]. In response to these challenges, researchers are now developing advanced delivery platforms, including miRNA-loaded nanoparticles and exosome-based carriers, which enhance targeting specificity, stability in circulation, and overall safety. These innovations mark important progress toward the integration of miRNA therapeutics into the framework of precision oncology [74,75,76].

While certain miRNAs, such as miR-21 and miR-155, are shared between CCA and PDAC, reflecting common oncogenic pathways and mechanisms of therapeutic resistance, distinct miRNA expression patterns also differentiate these malignancies. These differences are attributable to variations in tissue of origin, tumor microenvironment, and molecular pathogenesis [1,69,76]. Tumor-specific miRNAs regulate divergent oncogenic networks, offering mechanistic insight and facilitating the development of cancer-type–specific therapeutic approaches [82,83,84].

For instance, although both CCA and PDAC exhibit activation of the PI3K/AKT and inflammatory signaling pathways via shared miRNAs, PDAC is uniquely characterized by the dysregulation of miRNAs involved in desmoplastic stromal interactions. miR-196a, in particular, has been implicated in promoting tumor invasiveness and early recurrence, further highlighting its potential as a PDAC-specific biomarker [82,83,84].

Despite arising from distinct anatomical locations, CCA and PDAC share multiple molecular characteristics, most notably, the dysregulation of miRNAs. Among these, miR-21 stands out as one of the most extensively investigated and consistently upregulated oncomiRs in both malignancies. In PDAC, elevated levels of miR-21 are strongly associated with increased tumor cell proliferation, resistance to apoptosis, and chemoresistance, particularly to gemcitabine [77,84]. Similarly, in CCA, miR-21 overexpression correlates with aggressive tumor behavior, heightened metastatic potential, and reduced overall survival. Mechanistically, miR-21 promotes oncogenesis by targeting the tumor suppressor PTEN, leading to activation of the PI3K/AKT signaling cascade, which enhances cell survival, proliferation, and resistance to therapy [75,76,77,84].

Another miRNA frequently upregulated in both CCA and PDAC is miR-155. In PDAC, miR-155 facilitates tumor progression by downregulating tumor suppressor genes and modulating immune-related signaling pathways, contributing to an inflammatory tumor microenvironment [81,82,83,84]. It is also implicated in inflammation-driven carcinogenesis, particularly in the context of pancreatitis. In CCA, elevated miR-155 levels have been associated with increased cellular proliferation, invasiveness, and EMT, further reinforcing its role in driving tumor aggressiveness [75,76,80,81,82,83,84] (Table 4).

The overlapping and divergent expression patterns of miRNAs in CCA and PDAC underscore the complex interplay between shared oncogenic mechanisms and tissue-specific molecular pathways. This convergence highlights the translational value of miRNA profiling as a tool to improve diagnostic accuracy, guide therapeutic decision-making, and monitor treatment response. Integrating miRNA-based biomarkers into clinical workflows could significantly enhance early detection and enable more precise therapeutic stratification in both malignancies.

Moving forward, future research should prioritize large-scale, multicenter investigations to validate miRNA signatures and to support the development of miRNA-modulating therapies. The use of advanced delivery technologies, such as nanoparticles and exosome-based platforms, may facilitate targeted, safe, and effective administration of these agents, thereby advancing the application of miRNA therapeutics within precision oncology frameworks [84,85].

Circulating miRNAs are increasingly recognized as powerful non-invasive biomarkers across a broad spectrum of malignancies, with promising applications in early detection, prognosis, and treatment monitoring. Their remarkable stability in bodily fluids and disease-specific expression patterns make them particularly attractive candidates for liquid biopsy-based diagnostics [5,83]. Among them, miR-21 has been extensively investigated in breast, colorectal, and lung cancers, where elevated levels correlate with tumor progression and adverse clinical outcomes. Similarly, miR-155 has gained attention as a reliable prognostic marker in hematological malignancies, reflecting both disease aggressiveness and therapeutic response [83,84].

Extending these observations to gastrointestinal cancers, recent studies have underscored the diagnostic and prognostic relevance of circulating miRNAs in CCA and PDAC. Notably, miR-21, miR-210, and miR-221, among others, have been linked to tumor burden, metastatic potential, and patient survival. This broader recognition of circulating miRNAs across multiple tumor types reinforces their clinical utility and highlights the need for further validation in CCA and PDAC to strengthen early detection strategies and advance personalized therapeutic approaches [83,84].

Beyond oncogenic miRNAs, tumor-suppressive miRNAs such as miR-34a and miR-145 also play pivotal roles across diverse malignancies. miR-34a, a downstream effector of the p53 tumor suppressor pathway, is central to the regulation of cell cycle arrest, apoptosis, and senescence, and has been implicated in cancers including lung, prostate, and gastric carcinomas [85]. Likewise, miR-145 is frequently downregulated in colorectal and ovarian cancers, where its loss promotes tumor cell proliferation, migration, and invasion. Given their broad tumor-suppressive functions, both miR-34a and miR-145 are currently being explored as potential therapeutic targets. Their dysregulation represents a unifying hallmark of oncogenesis, while their specific contributions to CCA and PDAC pathogenesis further justify their consideration as integral components of emerging frameworks for miRNA-based diagnostics and therapeutics [5,85].

## 5. Conclusions

miRNAs have emerged as pivotal components in the expanding field of molecular oncology, offering both shared and disease-specific avenues to advance the clinical management of CCA and PDAC. Their incorporation into precision medicine frameworks holds significant potential to refine diagnostic accuracy, improve patient stratification, and enhance therapeutic outcomes. In CCA, miRNAs perform multifaceted roles across the spectrum of tumor biology, contributing to disease initiation, progression, and therapeutic resistance. They serve not only as biomarkers for early detection and prognostication but also as actionable therapeutic targets and modulators of treatment response. Notably, miRNAs such as miR-21 have been implicated in resistance to standard chemotherapies, including gemcitabine and cisplatin, underscoring their relevance in overcoming drug resistance and optimizing treatment efficacy.

Although CCA and PDAC display distinct miRNA expression signatures reflective of their divergent tissue origins and biological behavior, a subset of dysregulated miRNAs is shared between the two malignancies. These overlapping miRNAs commonly regulate key oncogenic pathways, including PI3K/AKT activation, EMT, and inflammation-related signaling, suggesting convergent mechanisms of tumorigenesis. This intersection offers opportunities for developing unified diagnostic panels and cross-applicable therapeutic strategies that may benefit both cancer types.

Collectively, miRNAs are increasingly recognized as critical regulators in the pathophysiology and therapeutic targeting of CCA. Their dual utility, as diagnostic biomarkers and therapeutic agents, positions them at the forefront of translational cancer research. While currently available targeted therapies represent a major step forward, the future of CCA management may reside in integrated therapeutic approaches that combine genetic and epigenetic modulation. Continued clinical validation and mechanistic investigation will be essential to fully harness the clinical potential of these small regulatory RNAs in combating these highly aggressive malignancies.

## Figures and Tables

**Table 1:** miRNAs and Therapeutic Potential in Cholangiocarcinoma.

miRNA	Function in CCA	Potential Therapy	Subtype Association
miR-21 [1,85]	Promotes proliferation, invasion, and chemoresistance by inhibiting PTEN and PDCD4.	Anti-miR-21 inhibitors to restore tumor suppression.	iCCA and eCCA
miR-221/222 [1,85]	Enhances survival and metastasis by targeting p27 and PTEN.	miR-221/222 inhibitors for reducing tumor growth.	iCCA
miR-155 [1,2,3]	Linked to inflammation-induced CCA, enhances invasion.	Anti-miR-155 strategies to suppress tumor progression.	iCCA
miR-210 [1,2,3,5]	Involved in hypoxia adaptation, increasing resistance to therapy.	Targeting miR-210 to improve response to chemotherapy.	iCCA
miR-34a [33,37,38]	Suppresses Notch and Bcl-2 pathways, reducing proliferation and inducing apoptosis.	miR-34a mimics (e.g., MRX34) to restore tumor suppression.	Both (more data in iCCA)
miR-200 family [39,64]	Inhibits EMT and metastasis.	miR-200-based therapy to prevent tumor spread.	eCCA

Abbreviations: cholangiocarcinoma (CCA); intrahepatic cholangiocarcinoma (iCCA); extrahepatic cholangiocarcinoma (eCCA); epithelial–mesenchymal transition (EMT); phosphatase and tensin homolog (PTEN); programmed cell death protein 4 (PDCD4).

**Table 2:** miRNAs involved in pancreatic cancer.

miRNA	Function in PC	Potential Therapy
miR-155 [65]	Regulates inflammation and tumor growth via STAT3 activation.	miR-155 inhibitors to reduce tumor-promoting inflammation.
miR-221/222 [61,62,63]	Suppresses tumor suppressors p27 and PTEN, promoting metastasis.	AntagomiRs targeting miR-221/222.
miR-10b [58]	Facilitates invasion and EMT.	Inhibitors to reduce metastasis.
miR-34a [33,37,38]	Induces apoptosis by targeting Notch and Bcl-2 pathways.	miR-34a mimics (e.g., MRX34, a clinical trial drug).
miR-200 family [39,64]	Inhibits EMT, reducing metastasis.	miR-200-based therapies to block tumor spread.

Abbreviations: pancreatic ductal adenocarcinoma (PDAC); epithelial–mesenchymal transition (EMT); phosphatase and tensin homolog (PTEN); B-cell lymphoma 2 (Bcl-2); neurogenic locus notch homolog protein (Notch); signal transducer and activator of transcription 3 (STAT3).

**Table 3:** Liquid Biomarkers for miRNA in pancreatic cancer.

miRNA Biomarker	Source (Blood, Urine, etc.)	Diagnostic Potential
miR-21 [62,63,64]	Blood, Pancreatic Juice	High levels correlate with poor prognosis.
miR-155 [65]	Blood	Associated with early-stage pancreatic cancer.
miR-210 [39,64]	Blood	Predicts hypoxia and aggressive tumors.

**Table 4:** MiRNAs dysregulated in PC and CCA.

miRNA	Cancer Type	Role	Primary Targets	Pathways Involved	Clinical Implications
miR-21 [1,69,76]	PC and CCA	Oncogenic	PTEN, PDCD4, PTPN14	PI3K/AKT, EMT	Promotes proliferation, invasion, chemoresistance; poor prognosis
miR-370 [71,72,73,85]	PC	Dual (context-dependent)	FOXM1, PTEN, TP53, TGFBR2	WNT/β-catenin, EMT, inflammation	Biomarker for diagnosis/prognosis; target for therapy
miR-34a [1,85]	PC and CCA	Tumor suppressor	Bcl-2, Notch, CDK6	Apoptosis, cell cycle	Therapeutic mimic (MRX34); suppresses growth and promotes apoptosis
miR-200 family [3,4,27]	PC and CCA	Tumor suppressor	ZEB1/ZEB2, TGF-β pathway	EMT, metastasis	Maintains epithelial phenotype; prevents metastasis
miR-145 [75,76]	PC and CCA	Tumor suppressor	c-Myc, MDM2	Cell proliferation, apoptosis	Downregulated in tumors; suppresses growth
miR-221/222 [75,76]	CCA	Oncogenic	p27, PTEN	Cell cycle, survival	Promotes invasion, proliferation
miR-155 [65]	CCA	Oncogenic	SOCS1, TP53INP1	Inflammation, immune evasion	Links inflammation to tumor progression
miR-129-5p [84]	PC and CCA	Tumor suppressor	WNT, PI3K/AKT/mTOR components	Signaling cascades, neuro-oncology	Potential biomarker; under investigation

Abbreviations: pancreatic cancer (PC); cholangiocarcinoma (CCA); epithelial–mesenchymal transition (EMT); phosphatase and tensin homolog (PTEN); programmed cell death 4 (PDCD4); protein tyrosine phosphatase non-receptor type 14 (PTPN14); forkhead box protein M1 (FOXM1); tumor protein p53 (TP53); transforming growth factor beta receptor 2 (TGFBR2); Wingless-related integration site/β-catenin signaling pathway (Wnt/β-catenin); cyclin-dependent kinase 6 (CDK6); zinc finger E-box binding homeobox 1/2 (ZEB1/ZEB2); transforming growth factor beta (TGF-β); cellular myelocytomatosis oncogene (c-Myc); mouse double minute 2 homolog (MDM2); suppressor of cytokine signaling 1 (SOCS1); tumor protein p53 inducible nuclear protein 1 (TP53INP1); mechanistic target of rapamycin (mTOR).

## Data Availability

Not applicable.
